# Preface-Virtual Special Issue (VSI) of Methods X entitled: “Advanced mass spectrometric analysis for environmental and food safety”

**DOI:** 10.1016/j.mex.2021.101349

**Published:** 2021-04-16

**Authors:** Damia Barcelo, Mira Petrovic

**Affiliations:** Catalan Institute for Water Research (ICRA-CERCA), Girona, Spain

With great pleasure we would like to announce this new Virtual Special Issue (VSI) of Methods X entitled: “Advanced mass spectrometric analysis for environmental and food safety”. It includes selected papers presented at the 16th workshop on emerging high-resolution mass spectrometry (HRMS) and LC-MS/MS applications in environmental analysis and food safety held as virtual participation during 15–16 October 2020 in Barcelona, Spain. In addition to the papers presented at this conference this selection added as well regular papers published in Methods X that are within the Aims & Scope of the workshop. In total we have been able to compile 20 papers, 10 out of them presented at the conference.

The list of papers attached to this VSI collection evaluate practical aspects and state of the art applications in the field of HRMS and LC/MS/MS such as:

- Advances in HRMS instrumentation and their applicability in environmental and food analysis for small and large molecules (proteomics)
- Advantages, comparison and complementarities of advanced MS instruments (tandem and hybrid) in the quantitative and qualitative determination of complex environmental and food samples
- Advanced sample preparation technologies for food and environmental analysis including on-line pre-column technology coupled to LC/tandem MS
- Large number of applications in environmental analysis, basically water and soil/ sediment, biota and food, like fruits and vegetables, juices and meat.
- Broad range of contaminants and their transformation products

Examples reported describe useful applications of HRMS and LC/MS/MSS including as well non target screening (NTS) for the trace determination of pesticides in vegetables, fruits fish tissue and groundwater, sugars and amino acids in honey, heterocyclic amines inn ruminant meats, artificial sweeteners in surface water, pharmaceutical residues in soil and water, benzophenone-type UV filters in water samples, perfluoralkyl substances (PFASs) in water as well as analysis of cyanotoxins in drinking water and natural toxins in seawater . Environmental proteomics applied to Escherichia coli samples were reported as well expanding the field of applications of this annual conference from small molecule to large molecule determination. As complementary to MS analysis, a variety of sample preparation technologies were as well described and are included in the papers of this collection, among them PLE, QuECChERS, large volume injection and rapid SPE.

That being said we would like to thank all authors who presented their papers at the Barcelona conference as well as regular contributions to Methods X in compiling such a timely VSI. Most importantly this VSI will be of help to analytical chemists, newcomers, PhD students and those senior researchers that consider HRMS and MS/MS as one of emerging challenges of the years to come. In short, we invite them to send their new analytical developments in this field to Methods X.

To this end we have the pleasure to announce the 17th Workshop on Emerging HRMS and LC/MS/MS in environmental analysis and food safety to be held in Ottawa, Canada, 14–15 of October this year 2021.The format of the workshop will be an hybrid one, combining virtual and in person participation. We expect to meet some of you there! The chairs of the conference are also planning to prepare another VSI of Methods X on the same topic.

Thanks for reading and stay safe and healthy!

